# Decadal variability and recent summer warming amplification of the sea surface temperature in the Red Sea

**DOI:** 10.1371/journal.pone.0237436

**Published:** 2020-09-17

**Authors:** Kamal Aldien Alawad, Abdullah M. Al-Subhi, Mohammed A. Alsaafani, Turki M. Alraddadi

**Affiliations:** 1 Marine Physics Department, Faculty of Marine Sciences, King Abdulaziz University, Jeddah, Saudi Arabia; 2 Weather Forecast Division, Sudan Meteorological Authority, Khartoum, Sudan; University of Vigo, SPAIN

## Abstract

Under climate change, regional Sea Surface Temperature (SST) changes are a crucial factor affecting marine ecosystems, which thrive only within a certain thermal limit. Thirty-seven years of monthly gridded Optimum Interpolation SST data from 1982 to 2017 were used to investigate the decadal variability of this parameter in the Red Sea during the summer season, in relation to large-scale climate variability. We identified a non-uniform warming trend beginning around the mid-1990s over the whole basin, with a predominant amplified warming over the northern half (0.04°C year^-1^), which is approximately four times higher than the global warming trend, but much weaker warming over southern end (0.01°C year^-1^). It was found that the Atlantic Multi-Decadal Oscillation (AMO) and the Silk Road Pattern (SRP) are shaping the RS SST, since their phase shift concurs with the timing of the significant non-uniform warming over the basin. The AMO triggers the SRP-related vertical and horizontal temperature advection that leads to opposite changes in the SST. We suggest that warming is amplified over the basin due to an overlap with global warming signals. Our results have important implications for interannual and decadal SST predictions based on the predictability of AMO and SRP patterns.

## 1. Introduction

The undoubted fact of climate change raises the potential for damaging effects on society, the environment and the overall ecosystem, with both the global mean Sea Surface Temperature (SST) and air temperature showing an increasing tendency in terms of frequency and intensity [1]. The SST’s increasing tendency is expected to continue even if global carbon dioxide and other greenhouse gases emissions remain constant or decrease [2]. However, the spatial distribution of the amplitudes of temperature increase are non-uniform [3]. The land is warming faster than the oceans [4–7], and mid and high latitude lands of the Northern Hemisphere are warming faster than the tropics due to the so-called polar amplification [8–10]. Within the ocean, some basins may be warming gradually; others may experience tipping points and rapid warming. Understanding the underlying mechanisms of these regional warming phenomena is crucial for recognizing the impacts on marine ecosystems, which also vary on the regional scale.

As such, the Red Sea’s (RS) vulnerability to SST warming is high, and associated with potentially harmful impacts on marine entities. Regionally, the warming process in the Indian Ocean, including the RS, may lead to thermal collapse [11]. Locally, Cantin et al. [12] and Roik et al. [13] have shown that the growth of central RS coral reefs is decreasing as the result of regional warming conditions. Meanwhile, the northern RS SST has been experienced a strong negative correlation with chlorophyll concentration [14, 15].

The long-term SST monthly climatology showed that August and February are the warmest and coolest months, with a significant trend of 0.5°C and 0.3°C decade^-1^, respectively [11]. The SST range between the summer and winter seasons is approximately 6°C [16]. Raitsos et al. [17] demonstrated that there was a shift in the annual mean SST from 27.4°C during 1985 to 1993, to 28.1°C during 1994 to 2007, associated with significant warming in the mid-90s. However, Hoegh-Guldberg et al. [11] observed there was an intense warming from 1982 to 2006. In addition, Chaidez et al. [18] found that the overall warming trend of the RS maximum SST is about 0.017°C year^-1^, while the northern RS alone warmed by between 0.04 and 0.045°C year^-1^ from 1982–2015, which all exceed the global rate of 0.011°C year^-1^. Furthermore, the authors noted increasing heat wave events in the northern half of the basin during the same period.

Recently, Shaltout [19] examined the warming of the RS SST from 1982 to 2016, and noted an intense warming trend of 0.029°C year^-1^. This warming is estimated to continue by an additional 0.6 to 0.85°C, based on the Intergovernmental Panel on Climate Change (IPCC)-Representative Concentration Pathway (RCP) scenario of 1.5 to 1.78°C (RCP4.5 scenario), 1.75 to 2.18°C (RCP6.0 scenario), and 2.8 to 3.28°C (RCP8.5 scenario), by the end of this century, using the GFDL-CM3 (Geophysical Fluid Dynamics Laboratory) model. However, intense warming trends were identified during the summer over Europe-Middle East (1.02°C) and Northeast Asia (1.08°C) that can be expressed as the difference in surface air temperature in 1997 to 2015 minus 1982 to 1996 [3, 20]. Furthermore, the warming extends to the lower middle troposphere as the result of the Atlantic Multi-Decadal Oscillation (AMO) shaping the summer surface and upper air temperature through the Silk Road Pattern (SRP) or the circumglobal teleconnection in some studies.

Due to the enormous economic value at stake and the large population affected, the warming over Europe and Northeast Asia has received widespread public attention. What has caused the RS warming is intriguing but remains largely understudied. To fill key knowledge gaps regarding the RS, the purpose of this study is to illustrate the possible mechanisms for these stronger warming trends.

The remaining part of this paper is organized as follows: Section 2 describes the datasets and methods used in this study. Section 3 presents the results. These results are discussed in Section 4 in which we explore how the SRP-related mechanism influences the RS SST. Finally, section 5 summarizes the main findings.

## 2. Data and methods

We used a gridded monthly Optimum Interpolation (OI) SST dataset (OISST; version 2) covering 1982 to 2017 in 0.25° grid points to investigate the decadal variability and recent warming in the RS. It merges satellite ocean skin temperatures and in situ observations from ships and buoys on a regular global grid, using the OI method to fill the gaps [21]. The OISST is the longest remotely sensed data from single instrument. With such high resoulution; several recent studies considered it relevant and appropriate dataset to study the local oceanic features, as well as in the semi-enclosed regional seas (eg, Nykjaer [22] in Medeterianian Sea and Shaltout [19] and Karnauskas and Jones [23] in the RS).

The monthly mean atmospheric circulation variables (including multiple-level temperature and wind) used in this study are from the ECMWF ERA-Interim re-analysis, conducted over the period of 1982–2017. Additionally employed is the 1982–2017 land-air surface temperature dataset (0.5° × 0.5°) from the Climate Research Unit (CRU), University of East Anglia, UK (version 4.02), that is based on weather station records [24]. The AMO index that defined as the annual averaged SST anomaly in the North Atlantic basin (0° to 60° N, 75° to 7.5° W) was obtained from www.esrl.noaa.gov/psd/data/timeseries/AMO/. The SRP is a teleconnection pattern trapped along the Asian upper-tropospheric westerly jet during summer, emanating from North Africa and propagating to East Asia across Europe roughly along 40° N. Following [3, 25] and Sun et al. [20], the SRP index is defined as the first leading principal component of the empirical orthogonal function for the 200 Geo-Potential Height (GPH) meridional wind anomalies within the domain (20°–60° N, 0°–150° E) over 37 summers (1982–2017). In this study, all of the datasets are calculated as the summer mean, which indicates the averages over June–July–August (JJA). Furthermore, correlation and regression analysis are used to investigate the non-uniform warming trend in the basin.

## 3. Results

**Fig 1:**
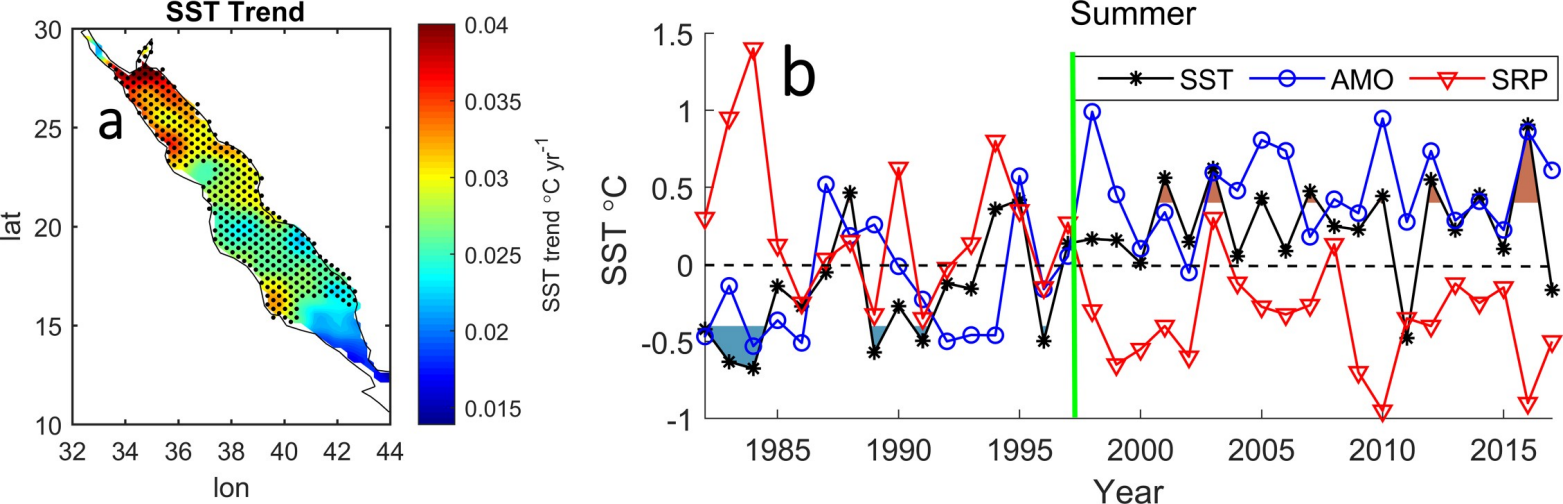
The spatial RS SST trend in °C year^-1^ during the summer season. The dot circle represents the 95% significance level (a). (b) the time series of the SST anomaly (black), AMO (blue) and SRP (red). The shaded area is the ±3 SST anomaly, while the vertical green line indicates the turning time of the three time series.

Fig 1A shows that the SST warming trend (1982–2017) is approximately 0.01°C year^-1^ in the south and 0.04°C year^-1^ in the northern end. The far northern end warming trend is approximately four times higher than the global warming trend (0.011°C year^-1^) from 1980 to 2005 [1]. It is clear from Fig 1B that the time series of SST during the summer season can be viewed as two distinct periods: the cold period (P1) from 1982 to 1996 with below average temperature, and the warm period (P2) from 1997 to 2017 with above average temperatures. Interestingly, these periods coincide with the warm (cold) phase of SRP during P1 (P2), respectively, and vice versa for the AMO. The thick green vertical line in Fig 1B indicates the shifting time between the two periods.

**Fig 2:**
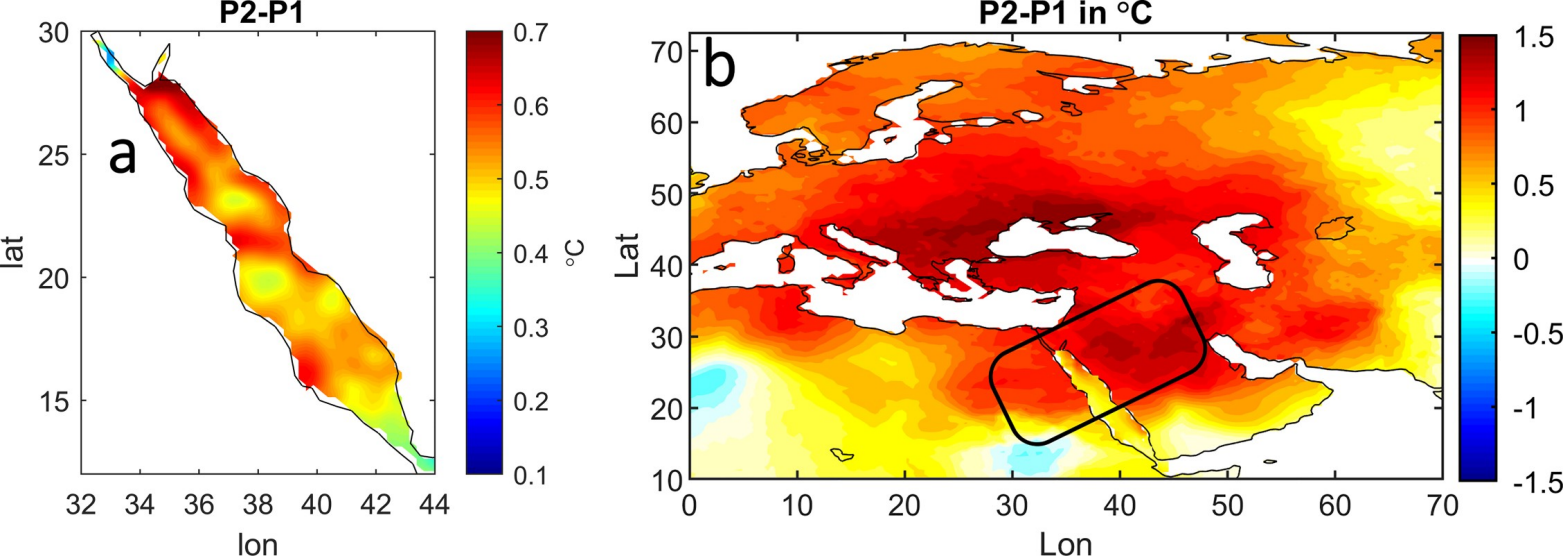
Difference in the summer SST in °C between P2-P1 (1997–2017 minus 1982–1996) (a). (b) as for (a) but for the surrounding land-air temperature.

Fig 2A shows the spatial differences in SST anomalies between the two periods (P2-P1); these two periods are used to identify the regional decadal changes in SST. Almost the entire basin has experienced a significant warming condition, but the most prominent warming spot is over the eastern side of the northern half (north 20° N), where the temperature increase is more than 0.5°C, and another spot on the eastern side of the southern RS. These spots make the figure look similar to the trend map in Fig 1A.

**Fig 3:**
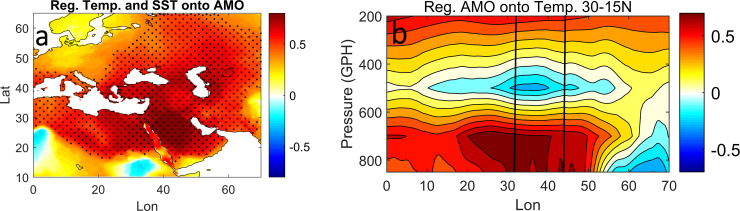
RS SST and surrounding land-air temperature regressed onto the AMO, the dot circle represent 95% significant level (a). While (b) the temperature at various pressure levels regressed onto the AMO. The two black vertical lines bound the area where the RS lies.

However, the intense warming is not limited to the RS, but also covers Europe and the Middle East, as can be seen in Fig 2B with an above 1°C temperature increase. In this analysis, we used the same data they used, but focused on the RS basin. In order to examine whether the modulation effect of the AMO on the air temperature can extend to the SST or not? we regressed the AMO index onto the RS SST and the surrounding land-air temperatures of the Europe-Middle East continent (Fig 3A).

Visually, the map looks similar to Fig 2B, the area of positive air temperature response to the AMO corresponds to the area of non-uniform warming (compare the boxes in Figs 3A and 2B).

For instance, the location of the RS surrounding area that has a strong positive relationship with the AMO (from the northern half of the RS to the Mediterranean Sea (20° to 30° N) and from the eastern RS to western Arabian Gulf) is the same area that shows intense warming in Fig 2B. Inside the RS, the SST exhibits a significant positive relationship with the AMO, which does not differ from the surrounding area in terms of relationship sign and intensity, especially in the northern half. Thus, the substantial consistency between the region of the amplified warming trend and the region of a positive AMO relationship with both the SST and surrounding air temperature implies that the AMO might play a role in inducing the non-uniform warming in the air and sea temperature [26]. This observation may lead to validation of the AMO mechanisms in shaping summer warming over Europe-Middle East through the SRP [3, 20], where the vertical temperature advection throughout the whole troposphere causes this warming.

Fig 3B shows the vertical temperature regressed onto the AMO along the RS from 15–30°N. In general, the vertically consistent warming in the lower troposphere corresponds to the area of non-uniform warming that surrounds the RS (compare the boxes in Fig 3A and 3B). For instance, the area of maximum positive relationship (up to 0.7) completely lies over the RS. However, there is a break in the mid-troposphere at 500-GPH, appearing as a weak negative relationship that extends zonally. In addition, the strong warming region (positive relation) seems to be confined below 700-GPH, which may imply the warming cannot be fully explained in terms of vertical advection from the upper troposphere. We repeated this analysis against the SRP instead of the AMO and it reflects similar results but with the opposite sign (not shown), which confirms our finding in Fig 1B that the time series of SRP and RS SST have opposite variability to each other.

**Fig 4:**
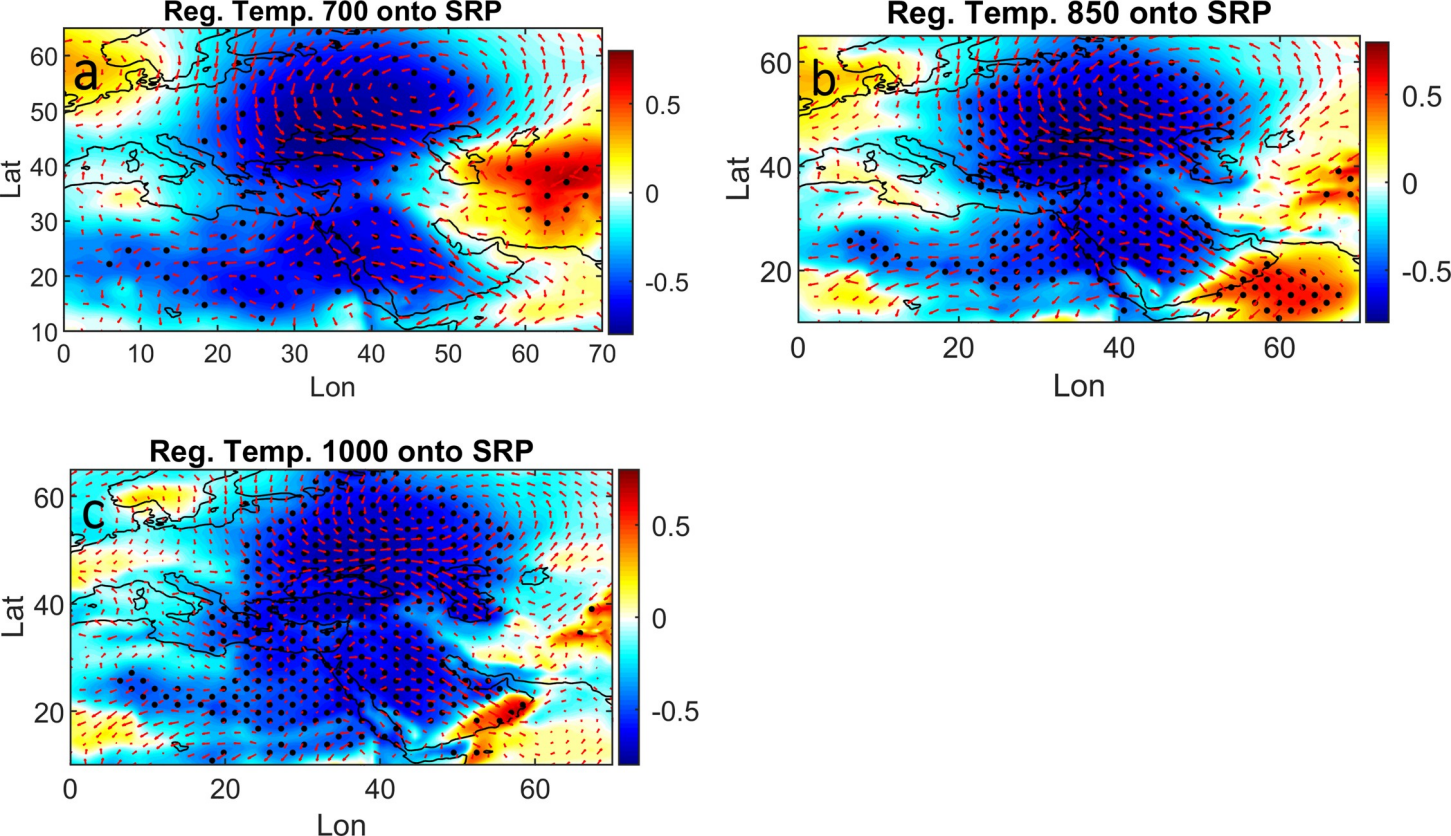
Temperature at 700 (a), 850 (b) and 1000 GPH (c), shaded color and wind (red arrows) regressed onto SRP, the dot circle represent 95% significant level.

The horizontal temperature advection is usually an important important factor for inducing change in both air and sea temperatures [27]. Fig 4 explores the SRP-related horizontal advection in terms of the temperature and zonal and meridional wind at various pressure levels. The regressions of 700,850 and 1000-GPH air temperature and horizontal wind onto the SRP show that the atmospheric circulation patterns over the whole Arabian Peninsula are associated with a steep gradient between Europe and the Arabian Peninsula. This kind of circulation in the lower troposphere allows advections of air masses toward the Arabian Peninsula, including over the RS. Since a wide part of Europe is experience non-uniform warming, it is not surprising to observe the northern half of the RS seems to have much warmer air than the southern part (Fig 2A and 2B). This is clear from the strong negative relationship (up to -0.8) over the northern basin that is associated with northerly wind at all levels (Fig 4A, 4B and 4C).

In brief, the regions of strong negative relationships in Fig 4 are consistent with the regions of warm SST and air temperature in Figs 2B and 3A, which might indicates the vital role of the AMO and SRP variability in influencing the RS SST and surrounding air temperature.

## 4. Discussion

The teleconnection through the atmosphere is a well-known topic relevant to understanding climate variability in the context of recent global warming. In this study, we investigated possible mechanisms for the amplified warming occurring over the RS SST during the summer season. We found that the significant warming that began in the mid-90s coincided with the onset of a positive phase of the AMO and negative phase of the SRP, exactly in 1997. Furthermore, the similarity between the SST trend map in Fig 1A and spatial differences in SST anomalies before and after 1997 (P2-P1) in Fig 2A implies that the AMO might be responsible for this significant warming through SRP-related atmospheric circulations. Comparable results were observed in the Mediterranean Sea where the long-term SST warming trend is highly correlated with the AMO [28].

Our analysis found that both vertical and horizontal advection are active, which allow the SRP footprint to appear in the RS. The contribution of vertical advection may be described through air subsidence. Usually, the northern RS and surrounding area exhibits a strong descending motion, while the southern RS exhibits a strong ascending motion, which result mainly from orographic lifting [29]. In this analysis, it was revealed that during positive AMO or negative SRP, the air subsidence of the lower to middle troposphere increases, which is conducive to increasing the adiabatic warming, increasing the aridity and hence amplifying the warming trend over the region. The SRP-related air subsidence is due to the anticyclonic circulations in the upper troposphere above the cyclonic circulation on the surface, over a wide part of Europe, the Eastern Mediterranean, and the Middle East and North African regions, causing reduced cloud cover and further precipitation, increased downward shortwave radiation, and then increased warming [20].

On other hand, the contribution of horizontal advection is also important, and may be described through the predominant wind system (Fig 4A–4C). From the regresion analysis of temerature and wind onto SRP over 700, 850 and 1000 GPH, the northerly wind was seen to be predominant in the lower and middle troposphere, associated with a strong negative relationship with the SRP.

Since the SRP phase turned from positive to negative in the mid-1990s (Fig 1B), the negative relationship implies that the warming might be amplified after the turning point and cover the Middle East and a wide part of Europe. Previous studies have recognized that the importance of this summer northerly wind is not confined to triggering the horizontal air mass advection to the Middle East. Besides that, it has broad social and environmental impacts and is locally called the Shamal Wind. For example, a strong Shamal caused a severe dust storm over the Arabian Peninsula [30–38]. The stationary anticyclonic system over the eastern Mediterranean, with a ridge extending toward northern Saudi Arabia, is responsible for the Shamal winds over the region [33, 39].

Furthermore, Al Senafi and Anis [40] identified that Shamal events cause abrupt changes in meteorological parameters during the summer season. For instance, an increase in wind speed, an increase in dust storms number,and an increase in temperature of 0.8°C have been observed, at the same time, a decrease in visibility and reduction in humidity. However, they identified that the number of summer Shamal days increased from 1997 to 2012 compared to 1973 to 1996, based on measurements from a station in Kuwait, which concurs with the phase shift of both AMO and SRP. These results may support our finding that the SRP, which is influenced by the AMO, induces different meteorological and oceanic variables in the Arabian Peninsula, including SST over the RS. Shamal winds bring drier air to the Arabian Peninsula while travelling over the continental land mass [40]. Since the central and northern part of the Arabian Peninsula is desert, the advected air masses toward the northern RS may become more drier and warmer. Based on the above, we are led to believe that this process may be amplifing the warming process that has developed over the region.

Furthermore, we explored whether these changes of RS SST and surounding air temperature between P1 and P2 affected the surface wind or not? We used the 10-meter surface wind from both era-interim and ERA5 datasets. The results does not show the expected change, which may refers to the difference in data type we used (re-analysis), while Al Senafi and Anis [40] used observed meteorological parameters from Kuwait.

We note a weak relationship of the temperature with the SRP on the pressure level over the southern RS, compared to the northern part. The orography features surrounding the southern part may be responsible for the weak relationship, since the mountainous area is characterized by ascending air masses and thus less warming. Attada et al. [29] explored the influence of the surface air temperature due to large-scale circulation patterns over the Arabian Peninsula, and showed that the lowest temperature values over the south-west Arabian Peninsula are related to orographic effects, in addition to its proximity to the cold Arabian Sea waters. Moreover, the upwelling conditions and open exchange with the Indian Ocean explain the slowed warming in the Gulf of Aden [19]. The above results follow the previous analysis by Belkin [41], who mentioned that the semi-enclosed nature of the northern half of the RS is responsible for its intensive warming trend.

Over all, these findings demonstrate the role of SRP in amplifying the warming in the RS SST. A question arises naturally: what influences the variation of SRP in interannual and decadal time scales? Previous studies have proposed various reasons, including subtropical heating anomalies over the Atlantic due to AMO forcing [3, 25], the forcing from central Africa, the Asian monsoon region, and the eastern Mediterranean Sea to the Caspian Sea region [42] and the Indian summer monsoon rainfall [43, 44].

It is important to note that amplified warming in the northern half of the RS may decrease the coral reef growth by decreasing its heat tolerance, since the maximum annual water temperatures are already close to coral thermal limits in the central RS [45]. Along same lines, a reduction in carrying capacity might occur where the SST is found to have a significant negative relationship with the chlorophyll-a concentration [14]. Considering that the AMO cycle is about 65–80 years [46] and it has been turned into a positive phase since the mid- 1990s, this indicates that strong warming will continue in the next decade. When these effects overlap with global warming signals, it causes amplified warming over the study area. These results have important implications for the interannual and decadal prediction of both the SST and air temperature patterns, based on the predictable cycle of the AMO and SRP.

In spite of these results, the role of large-scale tropical climate modes cannot be ignored, where the RS has direct water exchange with Indian Ocean. Furthermore, recent findings prove the great influence of the El Niño Southern Oscillation on sea level [47–49] and chlorophyll concentration [50] in the basin thorough oceanic and atmospheric teleconnections.

## 5. Conclusions

Based on disparate datasets from satellite and re-analysis, we emphasize the importance of the AMO footprint in shaping the summer non-uniform SST warming over the RS since the mid-1990s. This result is able to explain the current amplifying warming, and the most important findings of this study can be summarized as follows:

1. The timing of the significant non-uniform warming concurs with both the phase shift of the AMO and SRP time series, leading to an opposite change in the SST and surrounding land-air temperature.
2. The SRP time series experiences warm-1982 to 1996 (cold-1997 to 2017) phase that coincide with significant SST cooling (warming) over the RS and surrounding land-air temperature, including a wide part of Europe, the Eastern Mediterranean, and the Middle East and North Africa regions.
3. The mechanism by which this warming can be described is through vertical air subsidence from the middle to lower troposphere, as well as horizontal temperature advections in terms of the predominant wind system, which induce changes in both the sea and air temperatures surrounding the basin.
4. Accordingly, the warming is expected to continue into next decade, since the AMO and (SRP) have about a 65–80 years cycle, and they have been in a positive (negative) phase since the mid-1990s.

Our results demonstrate the increased possibility that warming conditions over the RS and in the surrounding area could make already hot places in the Arabian Peninsula unbearable for outdoor activities. It is also important for African and Arabian Peninsula fishermen to be aware of, due to alterations in marine ecosystems. Finally, our findings are of immediate importance in terms of implications for interannual and decadal predictions of the SST, and other physical and biological parameters in the RS.

## References

[1] IPCC, 2014. Climate Change 2013—The Physical Science Basis: Working Group I Contribution to the Fifth Assessment Report of the Intergovernmental Panel on Climate Change.; Cambridge University Press, Cambridge;

[2] Collins, M.; Knutti, R.; Arblaster, J.; Dufresne, J.-L.; Fichefet, T.; Friedlingstein, P., et al. Long-term climate change: projections, commitments and irreversibility. In Climate Change 2013-The Physical Science Basis: Contribution of Working Group I to the Fifth Assessment Report of the Intergovernmental Panel on Climate Change; Cambridge University Press, 2013; pp. 1029–1136.

[3] Hong X.; Lu R.; Li S. Amplified summer warming in Europe–West Asia and Northeast Asia after the mid-1990s. Environmental Research Letters 2017, 12, 094007.

[4] Sutton R.T.; Dong B.; Gregory J.M. Land/sea warming ratio in response to climate change: IPCC AR4 model results and comparison with observations. Geophysical Research Letters 2007, 34.

[5] Dong B.; Gregory J.M.; Sutton R.T. Understanding Land–Sea Warming Contrast in Response to Increasing Greenhouse Gases. Part I: Transient Adjustment. Journal of Climate 2009, 22, 3079–3097.

[6] Boer G.J. The ratio of land to ocean temperature change under global warming. Climate dynamics 2011, 37, 2253–2270.

[7] Joshi M.M.; Turner A.G.; Hope C. The use of the land-sea warming contrast under climate change to improve impact metrics. Climatic change 2013, 117, 951–960.

[8] Screen J.A.; Simmonds I. The central role of diminishing sea ice in recent Arctic temperature amplification. Nature 2010, 464, 1334 10.1038/nature09051 20428168

[9] Pithan F.; Mauritsen T. Arctic amplification dominated by temperature feedbacks in contemporary climate models. Nature Geoscience 2014, 7, 181.

[10] Xie S.-P.; Deser C.; Vecchi G.A.; Collins M.; Delworth T.L.; Hall A., et al Towards predictive understanding of regional climate change. Nature Climate Change 2015, 5, 921.

[11] Hoegh-Guldberg O.; Cai R.; Poloczanska E.S.; Brewer P.G.; Sundby S.; Hilmi K., et al Climate change 2014: impacts, adaptation, and vulnerability. Part B: regional aspects. Contribution of working group II to the fifth assessment report of the intergovernmental panel on climate change. 2014.

[12] Cantin N.E.; Cohen A.L.; Karnauskas K.B.; Tarrant A.M.; McCorkle D.C. Ocean warming slows coral growth in the central Red Sea. Science 2010, 329, 322–325. 10.1126/science.1190182 20647466

[13] Roik A.; Roder C.; Röthig T.; Voolstra C.R. Spatial and seasonal reef calcification in corals and calcareous crusts in the central Red Sea. Coral Reefs 2016, 35, 681–693.

[14] Eladawy A.; Nadaoka K.; Negm A.; Abdel-Fattah S.; Hanafy M.; Shaltout M. Characterization of the northern Red Sea’s oceanic features with remote sensing data and outputs from a global circulation model. Oceanologia 2017, 59, 213–237.

[15] Alawad K.A.; Al-Subhi A.M.; Alsaafani M.A.; Alraddadi T.M. Atmospheric Forcing of the High and Low Extremes in the Sea Surface Temperature over the Red Sea and Associated Chlorophyll-a Concentration. Remote Sensing 2020, 12, 2227.

[16] Berman T.; Paldor N.; Brenner S. Annual SST cycle in the eastern Mediterranean, Red Sea and Gulf of Elat. Geophysical Research Letters 2003, 30.

[17] Raitsos D.E.; Hoteit I.; Prihartato P.K.; Chronis T.; Triantafyllou G.; Abualnaja Y. Abrupt warming of the Red Sea. Geophysical Research Letters 2011, 38.

[18] Chaidez V.; Dreano D.; Agusti S.; Duarte C.M.; Hoteit I. Decadal trends in Red Sea maximum surface temperature. Scientific reports 2017, 7, 8144 10.1038/s41598-017-08146-z 28811521 PMC5557812

[19] Shaltout M. Recent sea surface temperature trends and future scenarios for the Red Sea. Oceanologia 2019.

[20] Sun X.; Li S.; Hong X.; Lu R. Simulated Influence of the Atlantic Multidecadal Oscillation on Summer Eurasian Nonuniform Warming since the Mid-1990s. Advances in Atmospheric Sciences 2019, 36, 811–822.

[21] Reynolds R.W.; Smith T.M.; Liu C.; Chelton D.B.; Casey K.S.; Schlax M.G. Daily high-resolution-blended analyses for sea surface temperature. Journal of Climate 2007, 20, 5473–5496.

[22] Nykjaer L. Mediterranean Sea surface warming 1985–2006. Climate Research 2009, 39, 11–17.

[23] Karnauskas K.B.; Jones B.H. The interannual variability of sea surface temperature in the Red Sea from 35 years of satellite and in situ observations. Journal of Geophysical Research: Oceans 2018, 123, 5824–5841.

[24] Harris I.; Jones P.D.; Osborn T.J.; Lister D.H. Updated high-resolution grids of monthly climatic observations–the CRU TS3. 10 Dataset. International journal of climatology 2014, 34, 623–642.

[25] Hong X.; Lu R. The meridional displacement of the summer Asian jet, Silk Road Pattern, and tropical SST anomalies. Journal of Climate 2016, 29, 3753–3766.

[26] Krokos G.; Papadopoulos V.P.; Sofianos S.S.; Ombao H.; Dybczak P.; Hoteit I. Natural climate oscillations may counteract Red Sea warming over the coming decades. Geophysical Research Letters 2019, 46, 3454–3461.

[27] Sun J.; Wang H.; Yuan W. Decadal variations of the relationship between the summer North Atlantic Oscillation and middle East Asian air temperature. Journal of Geophysical Research: Atmospheres 2008, 113 10.1029/2007jd009286 24383047 PMC3874577

[28] Skliris N.; Sofianos S.; Gkanasos A.; Mantziafou A.; Vervatis V.; Axaopoulos P., et al Decadal scale variability of sea surface temperature in the Mediterranean Sea in relation to atmospheric variability. Ocean Dynamics 2012, 62, 13–30.

[29] Attada R.; Dasari H.P.; Parekh A.; Chowdary J.S.; Langodan S.; Knio O., et al The role of the Indian Summer Monsoon variability on Arabian Peninsula summer climate. Climate Dynamics 2019, 52, 3389–3404.

[30] Yu Y.; Notaro M.; Kalashnikova O.V.; Garay M.J. Climatology of summer Shamal wind in the Middle East. Journal of Geophysical Research: Atmospheres 2016, 121, 289–305.

[31] Notaro M.; Yu Y.; Kalashnikova O.V. Regime shift in Arabian dust activity, triggered by persistent Fertile Crescent drought. Journal of Geophysical Research: Atmospheres 2015, 120, 10–229.

[32] Yu Y.; Notaro M.; Liu Z.; Wang F.; Alkolibi F.; Fadda E., et al Climatic controls on the interannual to decadal variability in Saudi Arabian dust activity: Toward the development of a seasonal dust prediction model. Journal of Geophysical Research: Atmospheres 2015, 120, 1739–1758.

[33] Hamidi M.; Kavianpour M.R.; Shao Y. Numerical simulation of dust events in the Middle East. Aeolian Research 2014, 13, 59–70.

[34] Notaro M.; Alkolibi F.; Fadda E.; Bakhrjy F. Trajectory analysis of Saudi Arabian dust storms. Journal of Geophysical Research: Atmospheres 2013, 118, 6028–6043.

[35] Yu Y.; Notaro M.; Liu Z.; Kalashnikova O.; Alkolibi F.; Fadda E., et al Assessing temporal and spatial variations in atmospheric dust over Saudi Arabia through satellite, radiometric, and station data. Journal of Geophysical Research: Atmospheres 2013, 118, 13–253.

[36] Goudie A.S.; Middleton N.J. Desert dust in the global system; Springer Science & Business Media, 2006;

[37] Shao Y. A model for mineral dust emission. Journal of Geophysical Research: Atmospheres 2001, 106, 20239–20254.

[38] Middleton N.J. A geography of dust storms in South-west Asia. Journal of Climatology 1986, 6, 183–196.

[39] Nasrallah H.A.; Nieplova E.; Ramadan E. Warm season extreme temperature events in Kuwait. Journal of Arid Environments 2004, 56, 357–371.

[40] Al Senafi F.; Anis A. Shamals and climate variability in the Northern Arabian/Persian Gulf from 1973 to 2012. International Journal of Climatology 2015, 35, 4509–4528.

[41] Belkin I.M. Rapid warming of large marine ecosystems. Progress in Oceanography 2009, 81, 207–213.

[42] Yasui S.; Watanabe M. Forcing processes of the summertime circumglobal teleconnection pattern in a dry AGCM. Journal of Climate 2010, 23, 2093–2114.

[43] Ding Q.; Wang B. Circumglobal teleconnection in the Northern Hemisphere summer. Journal of Climate 2005, 18, 3483–3505.

[44] Rodwell M.J.; Hoskins B.J. Subtropical anticyclones and summer monsoons. Journal of Climate 2001, 14, 3192–3211.

[45] Osman E.O.; Smith D.J.; Ziegler M.; Kürten B.; Conrad C.; El-Haddad K.M., et al Thermal refugia against coral bleaching throughout the northern Red Sea. Global change biology 2018, 24, e474–e484. 10.1111/gcb.13895 29044761

[46] Enfield D.B.; Mestas-Nuñez A.M.; Trimble P.J. The Atlantic multidecadal oscillation and its relation to rainfall and river flows in the continental US. Geophysical Research Letters 2001, 28, 2077–2080.

[47] Alawad K.A.; Al-Subhi A.M.; Alsaafani M.A.; Alraddadi T.M.; Ionita M.; Lohmann G. Large-Scale Mode Impacts on the Sea Level over the Red Sea and Gulf of Aden. Remote Sensing 2019, 11, 2224.

[48] Al-Subhi A.M.; Alsaafani M.A.; Alraddadi T.M. Signatures of Tropical climate modes on the Red Sea and Gulf of Aden Sea Level. 2017.

[49] Abdulla C.P.; Al-Subhi A.M. Sea Level Variability in the Red Sea: A Persistent East–West Pattern. Remote Sensing 2020, 12, 2090.

[50] Raitsos D.E.; Yi X.; Platt T.; Racault M.-F.; Brewin R.J.; Pradhan Y., et al Monsoon oscillations regulate fertility of the Red Sea. Geophysical Research Letters 2015, 42, 855–862.

